# MRI Findings of Muscle Damage after Total Hip Arthroplasty Using the Complete Muscle Preserving Anterolateral Supine Approach

**DOI:** 10.3390/medicina58060713

**Published:** 2022-05-26

**Authors:** Shuhei Oda, Takashi Hisatome, Eiji Cho, Hirohisa Fujimaki, Kazuyoshi Nakanishi

**Affiliations:** 1 Department of Orthopedic Surgery, Nihon University Hospital, 1-6, Kanda-Surugadai, Chiyoda-ku, Tokyo 101-8309, Japan; tome_westernlari@yahoo.co.jp (T.H.); cho.eiji@nihon-u.ac.jp (E.C.); fujimaki.hirohisa@nihon-u.ac.jp (H.F.); 2 Department of Orthopaedic Surgery, Nihon University Itabashi Hospital, 30-1, Ohyaguchikami-cho, Itabashi-ku, Tokyo 173-8610, Japan; nakanishi.kazuyoshi@nihon-u.ac.jp

**Keywords:** anterolateral total hip arthroplasty, muscle damage, atrophy

## Abstract

*Background and Objectives:* We performed anterolateral total hip arthroplasty (ALS THA) with the purpose of complete muscle-tendon preservation without muscle-tendon dissection. This study aimed to evaluate muscle damage in the periprosthetic hip joint muscles of patients undergoing ALS THA at 1-year post-operative hip magnetic resonance imaging (MRI). *Materials and Methods:* We evaluated changes in the muscle cross-sectional area (M-CSA) and fatty atrophy of the periprosthetic muscles. We also assessed the Harris hip score on pre-operative and 12-month post-operative MRI in 66 patients who underwent ALS THA. The grade of M-CSA atrophy was classified into no atrophy, slight atrophy, moderate atrophy, and severe atrophy. Fatty atrophy was classified as improved, no change, and worsened using the Goutallier classification. *Results:* More than 90% of patients’ M-CSA had no atrophy in the obturator internus (Oi), obturator externus (Oe), gluteus medius (Gmed), and gluteus minimus (Gmin), and some improvement was observed in terms of fatty atrophy. In contrast, M-CSA of the tensor fascia latae (TFL) muscle was clearly decreased, and there was no improvement in the TFL fatty atrophy. However, the presence or absence of TFL atrophy did not affect clinical outcome. *Conclusions:* We performed the complete muscle preserving procedure, ALS THA, with attention to preserving the Oi and Oe by direct visual confirmation and gentle treatment of the Gmed and Gmin with effective retraction. Post-operative M-CSA atrophy evaluation on MRI showed that the Oi, Oe, Gmed, and Gmin were satisfactorily preserved; however, the TFL was clearly atrophic. In the ALS approach, where entry is made between Gmed and TFL, atrophy of the TFL due to superior gluteal nerve injury must be tolerated to some extent.

## 1. Introduction

Anterolateral total hip arthroplasty, based on the Watson-Jones approach, is a technique that reduces soft-tissue involvement by entering through the intermuscular space between the gluteus medius (Gmed) and tensor fascia latae (TFL) muscles, thereby reducing soft-tissue invasion and allowing for a quicker recovery. The anterolateral approach is performed in the supine or lateral decubitus position. The approach performed in the supine position is called the anterolateral supine approach (ALS), and the one performed in the lateral position is called the Orthopädische Chirurgie München (OCM) approach, both of which involve entry from between the muscles of the Gmed and TFL. In the anterolateral approach, the superior gluteal nerve (SGN) runs between the Gmed and TFL, and SGN nerve damage may occur. The advantages of ALS THA include less post-operative pain, a lower dislocation rate, and easier intraoperative fluoroscopy. The disadvantages include tip fracture of the greater trochanter, the presence of a learning curve, and SGN injury. In addition, ALS THA has a learning curve, especially in femoral manipulation. Some centers that are unskilled in the technique may add a partial dissection of the short external rotator muscle for greater exposure. Therefore, there have been scattered reports of atrophy and fatty degeneration of the joint tendon on post-operative magnetic resonance imaging (MRI) evaluation after ALS THA [1,2]. In our hospital, we preserve the inferior band of the iliofemoral ligament (IBILFL) and the pubofemoral ligament. We detach only the ischiofemoral ligament at the lateral femoral attachment, so the conjoint tendon and the short rotator muscles can be seen in direct view. This surgical procedure allows for complete muscle-tendon preservation. In this study, we evaluated pre- and post-operative hip MRIs of ALS THA patients to measure changes in the muscle cross-sectional area (M-CSA) and fatty atrophy to investigate whether ALS THA with complete muscle-tendon preservation was achieved.

## 2. Materials and Methods

In this study, 66 patients with ALS THA at the same institution with 66 joints and unilateral osteoarthritis with normal contralateral sides were enrolled. Of these, 14 were men and 52 women, there were 35 right hip joints and 31 left hip joints, mean age (SD) was 68.5 (9.7) years, mean Body Mass Index (BMI) (SD) was 24.9 (1.8), and all patients had Crowe classification type I.

Total hip arthroplasty (THA) was performed in all patients under general anesthesia with complete muscle-tendon-sparing ALS THA by a single experienced surgeon using a cementless stem and cup with a tapered-wedge short stem. Intraoperative fluoroscopy was used to ensure accurate cup and stem placement. After surgery, all patients were allowed full weight-bearing the day after surgery and were discharged home when they could ambulate, ascend, and descend stairs. MRI was performed pre-operatively and 12 months post-operatively, and M-CSA of each muscle (obturator internus (Oi), obturator externus (Oe), Gmed, gluteus minimus (Gmin), and TFL) was measured on T1 imaged axial MRI, and the affected side/unaffected side ratio (A/N ratio) was calculated for each muscle. Atrophy was calculated using this formula: (post-operative patient tendon ratio/pre-operative patient tendon ratio) × 100%, where 75% or more was no atrophy, 50–75% was slight atrophy, 25–50% was moderate atrophy, and 25% or less was severe atrophy.

Fatty atrophy of the following muscles was assessed using the Goutallier classification (grade 0: no fat; grade 1: few fatty streaks; grade 2: < 50% fat; grade 3: 50% fat; grade 4: > 50% fat) [3]. Pre-operative and post-operative grades were compared. A change of 1 grade or more was considered a worsening or improvement of the muscle and was evaluated in three levels: improved, no change, and worsened. The Gmed and Gmin muscles were assessed at the anterior superior iliac spine level on axial T1-weighted MR images. The Oi, OE, and TFL were assessed at the lesser trochanter level. Pre- and post-operative M-CSA, fatty atrophy changes, and the Harris hip score were tested using a paired t-test. Multigroup comparisons of M-CSA and fatty atrophies in each muscle group were compared using the chi-square test and corrected using the Bonferroni test. Age, gender, BMI, the Harris hip score, and operative time were compared between normal and atrophic groups in the post-operative M-CSA for TFL using the unpaired Student’s t-test and chi-square test. For all tests, *p* < 0.01 was considered statistically significant.

### Surgical Technique of the Complete Muscle Preserving Procedure, ALS THA

**Figure 1:**
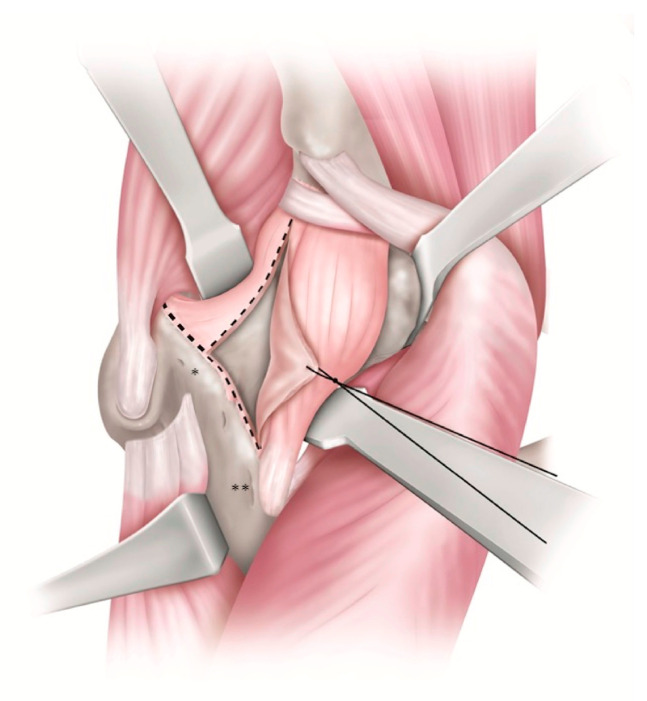
Inverted T-shaped incision of capsular ligament. The capsular attachment between the gluteus minimus (Gmin) and the anterior articular capsule ligament is detached. The inferior band of iliofemoral ligament (IBILFL) is preserved when possible; the inferior band is attached to the medial intertrochanteric eminence. The superior band of iliofemoral ligament (SBILFL) is attached to the lateral intertrochanteric eminence. *: Lateral intertrochanteric eminence **: Medial intertrochanteric eminence.

**Figure 2:**
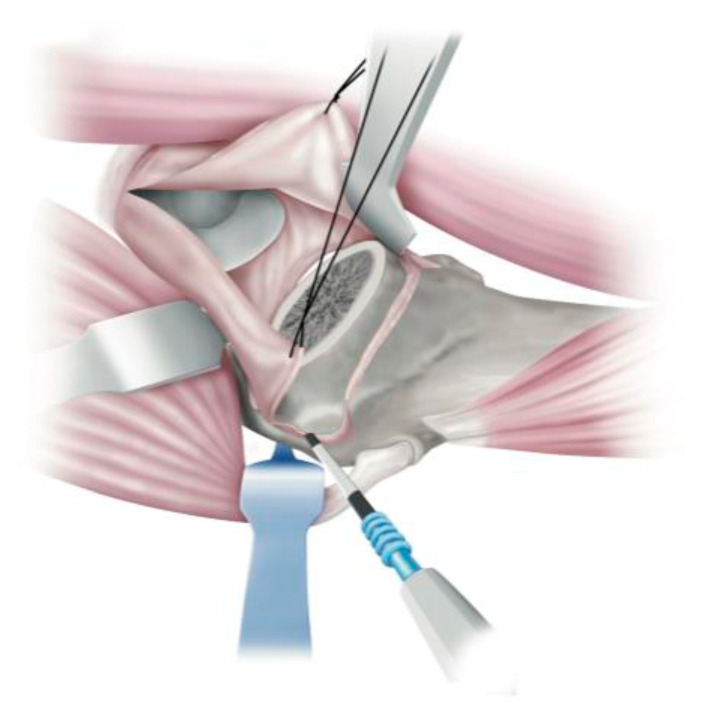
Incision of the superior band of the iliofemoral ligament. A Hohmann hook can be placed immediately proximal to the Gmin attachment site to prevent damage to the Gmin and effectively retract it. The superior band of the iliofemoral ligament (SBILFL) is incised over the lateral intertrochanteric eminence.

**Figure 3:**
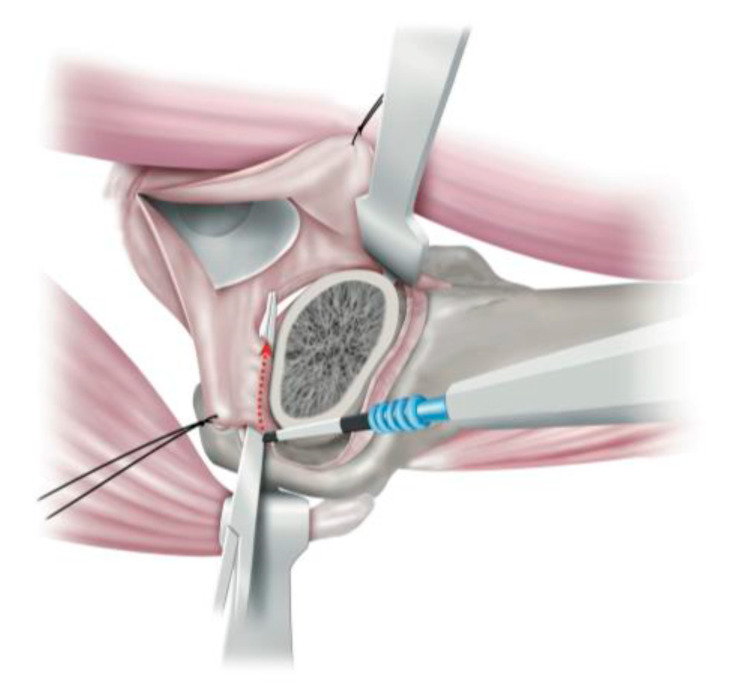
Incision of the ischiofemoral ligament.

**Figure 4:**
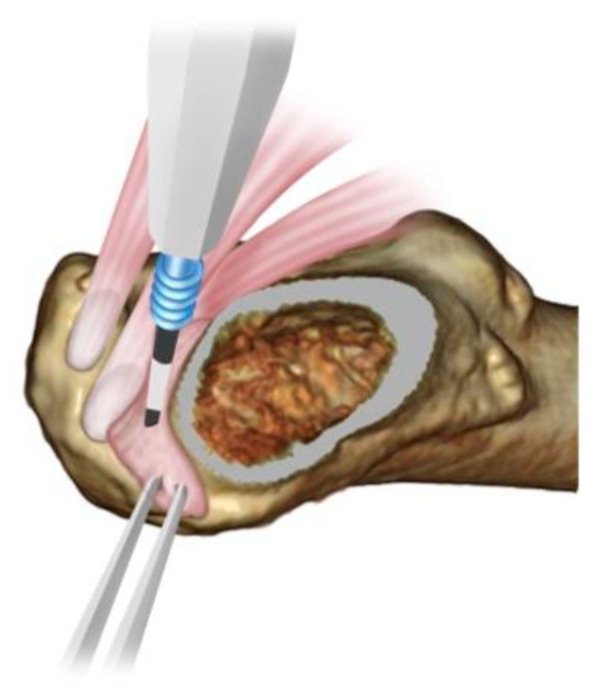
Resection of ischiofemoral ligament remnant. The conjoint tendon and Oe can be seen in direct view after resection of the ischiofemoral ligament remnant.

**Figure 5:**
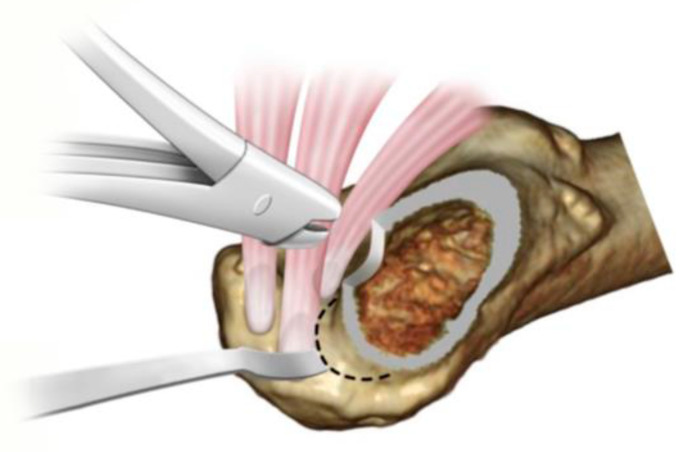
Osteotomy site at the femoral saddle. The conjoint tendon and Oe are identified under direct view and resect the bone in close proximity.

The surgery was performed with attention to the anatomical characteristics of muscles and ligaments, effectively retracting the muscles and confirming their preservation under direct vision. The surgeon also preserved and repaired articular capsule ligaments as much as possible to achieve appropriate muscle tension. The surgical technique is described in detail: the anterior articular capsule ligament was exposed, and the lateral ridge to which the superior band of the iliofemoral ligament (SBILFL) was attached was identified. During surgery, it was important to dissect the tight capsular attachment between the Gmin and bursa ligament. The capsular ligament was dissected through an inverted T-shaped incision while ensuring the preservation of the femoral attachment of the inferior band of iliofemoral ligament (IBILFL) (Figure 1). After resection of the femoral head, the SBILFL was separated from the lateral ridge where it was attached. The Gmin was locally attached to the anterior facet of the great trochanter. Insertion of the retractor proximally into the bald spot without damaging this attachment effectively exposed the anterior superior portion of the anterior articular capsule ligament (Figure 2). Forceps were inserted from the lateral side of the SBILFL into the anterior layer of conjoint tendons to protect the co-tendons and the external obturator tendon, with a dissection of the ischiofemoral ligament attachment with an electrocautery scalpel from inside the joint while protecting the conjoint tendon and the Oe (Figure 3). When all remnants of the ischiofemoral ligament left uncut were resected, the conjoint tendon and the Oe tendon were viewed under direct vision (Figure 4). The bone bordering those musculocutaneous tendons at the superior lateral saddle of the femoral neck was resected. Bone resection in this area allowed proper insertion of a reduced shoulder stem (e.g., tapered-wedge short stem) (Figure 5). Once the implant was in place, the partially transected articular capsule ligaments were sutured back together.

## 3. Results

**Figure 6:**
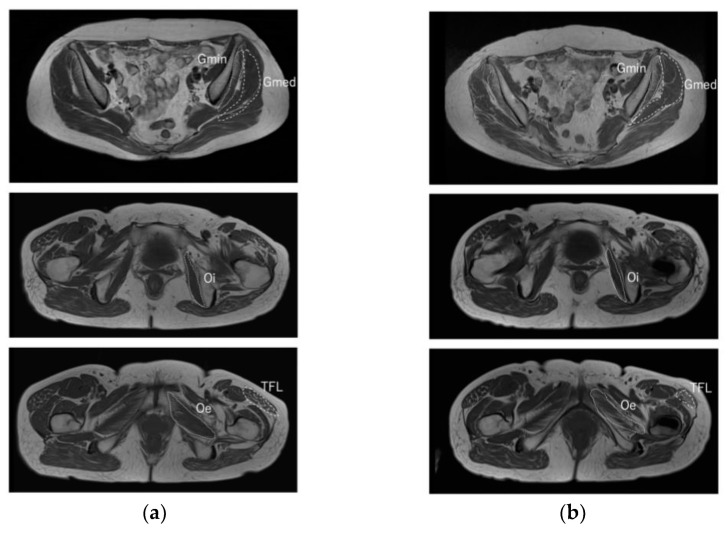
Pre-operative and 12-month post-operative T_1_-weighted magnetic resonance imaging axial scans of the bilateral hip joint. (**a**,**b**) show pre-operative and 12-month post-operative images, respectively. The left side is the affected side. Muscles surrounding the left hip joint analyzed were the Gmin (solid line), the Gmed (dashed line), the Oi (solid line), the Oe (dotted line), and the tensor fascia latae (TFL) (dashed line). Post-operative TFL shows decreased muscle cross-sectional area and worsened fatty atrophy.

The affected side/unaffected side ratio (A/N) ratio of the M-CSA showed no obvious pre- and post-operative changes in the Oi and Oe; however, there was a significant post-operative decrease in the Gmed, Gmin, and TFL (Table 1). On the only affected side, pre-operative and post-operative M-CSA were compared: there was no significant post-operative atrophy for Oi and Oe, but there was significant post-operative atrophy for Gmed, Gmin, and TFL. These results were similar to the results of M-CSA using the A/N ratio (Table 2). More than 90% of cases were included as having no atrophy in Oi, Oe, Gmed, and Gmin in the M-CSA atrophy assessment (Table 3). In contrast, 47 joints (71%) in the TFL were no atrophy, 16 joints (24%) were slight atrophy, and 3 joints (5%) were moderate atrophy, clearly indicating more atrophy in TFL. A significant difference was observed in the multigroup comparison (*p* < 0.0001) (Figure 6). Fatty atrophy grade was not significantly different in the Oi, Oe, Gmed, and Gmin muscles before and after surgery; however, it was significantly lower in TFL (*p* = 0.0063) (Table 4). Fatty atrophy changes were as follows: for Oi, 6 (9.1%) worsened, 58 (87.9%) had no change, and 2 (3.0%) improved; for Oe, 8 (12.1%) worsened, 55 (83.3%) had no change, and 3 (4.6%) improved; for Gmed, 7 (10.6%) worsened, 57 (86.4%) had no change, and 2 (3.0%) improved; for Gmin, 9 (13.6%) worsened, 53 (80.3%) had no change, and 4 (6.1%) improved; for TFL, 8 (12.1%) worsened, 58 (87.9%) had no change, and 0 (0%) improved; and no significant differences were observed in the multigroup comparisons of each muscle (*p* = 0.95) (Table 5). The Harris hip score was 46.5 ± 0.94 pre-operatively and 91.1 ± 0.71 post-operatively, and the patients showed a significant improvement (*p* < 0.0001). The TFL patients were divided into two groups—one with more than slight atrophy and one without atrophy—and were compared in terms of age, gender, BMI, and pre- and post-operative Harris hip scores. No significant differences were observed between both groups (Table 6).

## 4. Discussion

Posterior, direct lateral, anterolateral, and direct anterior approaches have been established for THA. Damage to Gmed, Gmin, short external rotator muscles, and TFL usually depends on the surgical approach. In the posterior approach, the short external rotator is usually detached from its attachment and reattached to the non-anatomic insertion. However, a high detachment rate of the attachment site has been reported [4].Pellicci Pellici et al. evaluated 30 cases in which the short external rotators were repaired by the THA posterior approach using MRI at 3 months post-operatively and reported that 17 of 30 cases (57%) showed a dissection of 25 mm or more at the conjoint tendon [5]. Recently, direct anterior (DAA) and anterolateral approaches, which are muscle-sparing anterior approaches, have become popular due to their low dislocation rates. Compared to the direct lateral and posterior approaches, less soft tissue damage in this approach has been reported [6]. However, there have been scattered reports of muscle atrophy after DAA. Kawasaki et al. reported that MRI evaluation after DAA showed damage to the Gmin, TFL, and OI, especially in developmental dysplasia of the hip (DDH) cases, causing severe damage to the OI [7]. DAA requires more anterior elevation of the femur than ALS; hence, it is especially common for the OI to be debrided. The ALS approach is the first choice for DDH cases in our institution. Unlike DAA, ALS requires external migration of the femur. The femur must be held in adduction and over 90 degrees of external rotation to allow the insertion of the femoral stem, which requires dissection of the ischiofemoral ligament attachment. We prevent damage to the OI by ensuring that the ischiofemoral ligament is dissected. The key to a better expansion of the surgical field is the effective retraction of the Gmed and Gmin. By confirming OI and OE, muscle damage can be minimized. Reportedly, the anterior approach is more likely to damage the posterior portion of the intermuscular space (in DAA, TFL is the posterior component, and in ALS, Gmed and Gmin are the posterior components in the surgical approach) [8,9]. Our study showed that the A/N ratio of the M-CSA was decreased in the Gmed and Gmin. The possible cause of this was that although the Gmed and Gmin were identified and retracted under direct observation, they may have been damaged by the direct pressure of the retractor due to the intraoperative femoral elevation maneuver. However, over 75% of the muscle volume was maintained in more than 90% of the patients, suggesting minimal muscle damage due to intraoperative manipulation.

The ALS THA is an approach which utilizes the interval between the Gmed and TFL; however, this muscle space is not a true internervous plane. It has long been noted that entry from this intermuscular space results in SGN injury. Several travel patterns of the descending branches of the superior gluteal nerve have been described [10]. In a study by Vasco et al. [11], the average distance to the greater trochanter at the site where the peripheral motor branches of the SGN entered the TFL was 72.5 mm, with a large range of 40–110 mm. They also observed a considerable variation in the branching pattern of nerves before entering the TFL and defined the “red zone” as the region where 75% of the SGN terminal branches entered the TFL. In ALS THA, an arthrotomy from the anterior inferior iliac spine to the lateral ridge is essential to incise the anterior iliac spine and enter the joint. To obtain that field of view, about 40 mm from the apex of the greater trochanter must be developed proximally between the Gmed and TFL. This is within the red zone proposed by Vasco [11], who considered that the terminal branches of the descending SGN branches running to the TFL are more likely to be damaged in the ALS THA approach. Unis et al. [12] observed TFL atrophy in 61% of 16 patients in 26 THAs using a modified anterolateral approach. Fatty degeneration was seen in 11 patients (42%), and overall, 74% of patients showed TFL atrophy or hypertrophy on MRI at a median of 9.3 months. Contrarily, Takada et al. [1] evaluated 164 patients who underwent cementless THA with a modified anterolateral approach on pre- and post-operative MRI and reported a low incidence of TFL atrophy in 13 patients (8.0%). However, they defined TFL atrophy as a deterioration of two or more steps in the Goutallier classification or a decrease in the cross-sectional area of the TFL by 40% or more, which seems to be an underestimation because the criteria for atrophic change are too strict. Grob et al. [13] stated that the diagnosis of SGN terminal branch lesions may be delayed because they are not always accompanied by symptoms. They reported that a patient who underwent THA via an anterior approach had a depressed TFL that was visibly atrophic from the outside but had exactly the same good clinical and functional results as the contralateral side. In this study, there was no significant difference in the post-operative clinical outcomes between the atrophied and non-atrophied groups of the TFL, which may not be a major clinical obstacle. In the ALS approach, which utilizes the interval between Gmed and TFL, there is a high risk of damage to the TFL branch, especially when deploying proximally. However, it is difficult to predict branching patterns in advance, and considering the overall benefits of ALS THA, we believe that some damage to the SGN branches must be tolerated.

This study had some limitations. In our study, we evaluated MRI at 1 year post-operatively, and we were unable to track MRI changes beyond that time. Ismailidis et al. [14] stated that hip abductor muscle weakness might gradually improve during the first 24 months after THA. It is possible that M-CSA atrophy and fatty atrophy in MRI may resolve in the future when MRI is followed for more than one year. Second, the MRI horizontal stage was used to evaluate the images in two dimensions but not in three dimensions. A more detailed evaluation could have been carried out by conducting the evaluation in three dimensions. Third, our study did not include interobserver variability in the assessment of muscle atrophy and fatty atrophy. As for the TFL atrophy, we considered that it was due to the SGN injury; however, it could have been directly injured during intraoperative deployment or femoral manipulation. A combination of MRI and electromyography may be effective in resolving this issue.

## 5. Conclusions

Post-operative muscle damage in ALS THA was evaluated by MRI. ALS THA with a complete muscle-preserving procedure performed under direct visualization with OI and OE preservation was observed to reduce muscle damage to OI and OE. The retraction maneuver during femoral elevation caused minor damage to the Gmed and Gmin; however, muscle volume was preserved in most patients. Furthermore, 29% of TFLs had muscle atrophy thought to be caused by SGN injury. In the anterolateral approach, TFL atrophy due to SGN injury may occur to some extent.

## Figures and Tables

**Table 1:** A/N ratio of muscle cross-sectional area pre-operatively, 12 months post-operatively.

	Pre-Operatively	12 Months	Statistics
Oi	0.94 (±0.0089)	0.92 (±0.010)	*p* = 0.13
Oe	0.95 (±0.0098)	0.92 (±0.018)	*p* = 0.19
Gmed	0.94 (±0.0055)	0.86 (±0.012)	*p* < 0.0001
Gmin	0.87 (±0.010)	0.78 (±0.015)	*p* < 0.0001
TFL	0.90 (±0.017)	0.71 (±0.025)	*p* < 0.0001

A/N ratio, affected side/unaffected side ratio;Oi, obturator internus; Oe, obturator externus; G med, gluteus medius; G min, gluteus minimus; TFL, tensor fascia latae.

**Table 2:** Affected side of muscle cross-sectional area pre-operatively and 12 months post-operatively.

	Pre-Operatively	12 Months	Statistics
Oi	769.4 (±19.9)	758.8 (±20.0)	*p* = 0.36
Oe	1163.6 (±41.0)	1137.2 (±41.5)	*p* = 0.27
Gmed	2203.9 (±54.6)	2059.1 (±58.0)	*p* = 0.0002
Gmin	880.1 (±26.3)	804.2 (±24.9)	*p* = 0.0002
TFL	530.8 (±20.1)	449.9 (±23.6)	*p* = 0.0001

**Table 3:** Post-operative A/N ratio of M-CSA atrophy grade of each muscle.

	Oi (%)	Oe (%)	Gmed (%)	Gmin (%)	TFL (%)
No atrophy: >75%	62 (94%)	63 (95%)	62 (94%)	61 (92%)	47 (71%)
Slight atrophy: 50–75%	4 (6%)	3 (5%)	4 (6%)	5 (8%)	16 (24%)
Moderate atrophy: 25–50%	0 (0%)	0 (0%)	0 (0%)	0 (0%)	3 (5%)
Severe atrophy: <25%	0 (0%)	0 (0%)	0 (0%)	0 (0%)	0 (0%)

**Table 4:** Fatty atrophy grade of each muscle pre-operatively, 12 months post-operatively.

	Pre-Operatively	12 Months	Statistics
Oi	0.17 (±0.51)	0.26 (±0.062)	*p* = 0.057
Oe	0.71 (±0.064)	0.83 (±0.059)	*p* = 0.059
Gmed	1.24 (±0.057)	1.33 (±0.069)	*p* = 0.057
Gmin	2.21 (±0.079)	2.26 (±0.090)	*p* = 0.47
TFL	1.36 (±0.060)	1.55 (±0.081)	*p* = 0.0063

**Table 5:** Post-operative fatty atrophy change of each muscle.

	Oi (%)	Oe (%)	Gmed (%)	Gmin (%)	TFL (%)
Improved	2 (3%)	3 (5%)	2 (3%)	4 (6%)	0 (0%)
No change	58 (88%)	55 (83%)	57 (86%)	53 (80%)	58 (88%)
Worsened	6 (9%)	8 (2%)	7 (11%)	9 (14%)	8 (12%)

**Table 6:** Patient demographics and clinical scores in TFL atrophy and no atrophy.

	TFL Atrophy	No TFL Atrophy	Statistics
Number	19	47	N/A
Age	68.5 (±9.6)	68.6 (±8.2)	*p* = 0.97
Gender (Female/Male)	14/5	37/10	*p* = 0.91
BMI	25.5 (±3.0)	25.0 (±2.9)	*p* = 0.53
Pre-operative Harris hip score	48.0 (±7.9)	46 (±7.5)	*p* = 0.34
Post-operative Harris hip score	91.9 (±2.3)	90.7 (±6.7)	*p* = 0.43
Operative time (min)	97.7 (±5.5)	95.4 (±6.6)	*p* = 0.19

## Data Availability

The data presented in this study are available on request from the corresponding author. The data are not publicly available due to regulations of the local institutional ethics board.
